# Clinically Symptomatic Pericardial Effusions in Hospitalized Systemic Sclerosis Patients: Demographics and Management

**DOI:** 10.1155/2018/6812082

**Published:** 2018-06-04

**Authors:** Hitomi Hosoya, Chris T. Derk

**Affiliations:** ^1^Department of Medicine, Pennsylvania Hospital, Philadelphia, PA, USA; ^2^Division of Rheumatology, University of Pennsylvania, Philadelphia, PA, USA

## Abstract

**Background:**

Pericardial effusions in systemic sclerosis (SSc) may present as acute or chronic with or without clinical symptoms. Best treatment is unknown and whether patients receive medical therapy or a surgical procedure is clinician-dependent.

**Objective:**

To describe the clinical characteristics, treatment, and outcomes of patients with SSc and clinically symptomatic pericardial effusions treated in the inpatient setting.

**Methods:**

We evaluated all SSc admissions over a 10-year period to a tertiary care hospital which has a dedicated SSc clinic. Patients who had a clinically symptomatic pericardial effusion were evaluated based on their demographics, disease pattern, and medical or surgical management.

**Results:**

From January 2005 till October 2015, there were 462 SSc admissions with 32 (6.9%) of them being for a clinically symptomatic pericardial effusion in 23 unique patients. Eleven (47%) of these patients had right heart failure, seventeen (74%) had pulmonary arterial hypertension (PAH), and 4 (17%) had tamponade physiology. Five (22%) patients were treated by a surgical procedure, while eighteen (78%) patients had medical therapy. Patients who received medical therapy tended to be older, have a lower serum Cr level, and more likely have right heart failure.

**Conclusion:**

Clinically symptomatic pericardial effusion is a rare cause for hospital admissions in SSc, with a high percentage of these patients having PAH. Medical therapy tends to be reserved for older patients with right heart failure, while surgical therapy was more likely in patients with higher serum Cr levels.

## 1. Introduction

Systemic sclerosis (SSc) is an autoimmune rheumatic disease characterized by visceral and skin fibrosis, vascular dysfunction, and immune dysregulation. Cardiac involvement in SSc has been suggested in 15% to 35% of patients [1], though this is highly variable depending on the diagnostic technique used, that being postmortem pathology, echocardiographic or other imaging studies, and clinical presentation. Cardiac manifestations include myocardial (myocardial fibrosis, diastolic dysfunction, systolic dysfunction, and myocarditis), conduction system disease (heart block, autonomic dysfunction, supraventricular dysrhythmia, and ventricular dysrhythmia), and vascular (mural fibrosis, intimal proliferative, and platelet fibrin clots) [2]. Pericardial involvement ranges from 33% to 72% of SSc patients based on autopsy series [3–7] while in echocardiographic studies it ranges from 15% to 43% [8–10]. Pathologically it includes fibrinous pericarditis, chronic fibrous pericarditis, pericardial adhesion, and pericardial effusions [7, 11]. Clinically symptomatic pericardial effusions are present in only 5% to 16% of SSc patients [10–13].

Large pericardial effusions or cardiac tamponade are very rare in SSc and at times can even manifest prior to skin involvement. Large pericardial effusions are associated with a poor prognosis with a mortality rate of up to 55% [14, 15]. In most cases, pericardial fluid analysis shows an exudate while pulmonary hypertension is often a concomitant diagnosis [14].

Pericardial disease in SSc patients is usually treated with medical therapy such as corticosteroids, aspirin, NSAIDs, and colchicine, or in some cases surgical procedures such as a pericardiectomy or a pericardial window. Although pericardial disease leads to increased morbidity in systemic sclerosis, there is limited data on the outcomes of these treatments and the decision-making in regard to the specific therapies. With this study we attempt to characterize clinically symptomatic pericardial effusion management in hospitalized SSc patients and the predictors for the individual therapies.

## 2. Methods

After receiving institutional review board approval, we performed a single-site, retrospective study on patients with SSc and acute pericardial effusions who required hospitalization. Subjects were selected from all the patients who were admitted to the hospital of the University of Pennsylvania from January 1, 2005 to October 30, 2015. ICD- 9 codes for SSc and pericardial disease were used to identify the subjects. The diagnosis of SSc was confirmed as well as the diagnosis of the specific pericardial disease by detailed chart review. Data collected included the age, sex, race, type of SSc, SSc related characteristics such as Raynaud's, digital ulcers, interstitial lung disease, and esophageal involvement. Presenting clinical symptoms, laboratory studies, and echocardiographic results was also recorded, and readmission within 90 days and death up to February 2017 was also documented. Continuous variables were described as a mean with a standard deviation. Group differences among the patients who received medical therapy versus the patients who received surgical care were analyzed using Student's *t*-test as well as the Chi-square test for dichotomous variables.

## 3. Results

We identified 462 admissions with a diagnosis of SSc. Among them, there were 32 (6.9%) admissions of 23 individual patients with clinically symptomatic pericardial effusions. The mean age of this cohort was 60 (range: 44–79) years of age, with 19 (83%) female and 4 (17%) male patients. Fourteen patients were Caucasian (61%), 5 African American (22%), 2 other, and 2 unknown. Eleven (47%) patients from the cohort had right heart failure and four (17%) had tamponade, while pulmonary arterial hypertension (PAH) was seen in 17 (74%) patients of our cohort. From the 23 patients evaluated 5 (22%) ended up receiving a pericardial surgical procedure during their admission to the hospital (Table 1). Clinical features and procedures are shown in Table 2. Five patients were newly diagnosed with SSc during the hospitalization. Five patients had overlap syndrome. Relevant medications prior to admission are listed. Out of 23 patients, 18 received medical therapy and 5 patients received a surgical procedure. The procedure group consisted of 3 patients who received a pericardiocentesis with a subsequent pericardial window, 1 patient who received only a pericardiocentesis, and 1 who received only a pericardial window. All patients in the procedure group had diffuse type of SSc while in the nonprocedure group 6 patients had limited type and 1 had diffuse while in the rest the type of SSc was not reported.

The mean age was 51.8 (SD 9.2) for the procedure group and 62.1 (SD 7.1) for the medical treatment group (*p* = 0.01). The main symptoms on presentation were shortness of breath (3 out of 5 in procedure group, 12 out of 18 in the medical group), chest pain (0 in procedure, 2 in medical), and fever (0 in procedure, 2 in medical). None had hypoxia on presentation in the procedure group while 7 did in the medical group. White blood cell (WBC) count, hemoglobin (Hgb), platelet (Plt), and blood urea nitrogen (BUN) were not significantly different between the groups. Creatinine was found to be significantly higher in the surgically treated group as compared to the medically treated group (*p* = 0.03). Pro-b-type natriuretic peptide (pro-BNP) tended to be higher in the medical group; however there was no statistically significant difference likely due to the small sample size. ANA was reported in 12 patients in the medical group; among them 10 were positive. In the procedure group, 5 were reported and 4 were positive. Scl-70 antibodies were done in 12 of the medical group patients and 4 of the procedure group and they were all negative. RNA polymerase III antibodies were not performed in any of the patients. Notably, the size of the pericardial effusion tended to be larger in the procedure group; however 5 patients in the medical group were recorded with “large” effusion on echocardiography. Three patients in the procedure group and 6 patients in the medical group had pleural effusion shown on a computed tomography (CT) of the chest. Eleven patients from the medical group had right heart failure while none did in the procedure group. Fourteen patients (78%) in the medical group and 3 (60%) in procedure group had pulmonary artery hypertension, and the mean pulmonary artery systolic pressure estimated by echocardiography was 79.8 ± 45 mmHg in the medical group and 39.7 ± 11 mmHg in the procedure group (*p* = 0.06). There were 2 patients each with tamponade physiology in both groups (Table 3).

For treatment, 7 out of 18 patients in the medical group and 3 out of 5 patients in the procedure group were treated with steroids (either oral or IV). The mean dosage of glucocorticoid given to the patients was 103 mg prednisone equivalent per day for medical therapy group and 345 mg for procedure group. Colchicine was administered to one patient in each group. Ten patients in the medical group received diuretics while 3 did in the procedure group. Two patients in the procedure group received azathioprine and cyclophosphamide, respectively. The results of fluid studies are included in Table 2. Pathology of pericardium was available for 3 patients in procedure group, one with chronic fibrosis with granulation tissue, another one with macrophage and reactive mesothelial cell without inflammatory cell infiltrate nor granulomas, and the other with normal appearance.

Two patients in the medical group died during the admission. Within 90 days of discharge, 3 readmissions occurred in the medical group and 1 in the procedure group, receiving a pericardiocentesis. This patient subsequently received a pericardial window. The outpatient charts were evaluated up to February 2017 and five more patients in the medical group and one in the procedure group died during followup.

## 4. Discussion

The present study describes the clinical manifestations, treatment, and outcomes of SSc patients who were admitted to the hospital with a clinically significant pericardial effusion. The decision-making for procedures in this group did not necessarily depend on the size of the pericardial effusion; while the two groups are small, some statistical comparisons were significant and in some cases there were some apparent trends among the two groups. The group that received medical therapy tended to be older (*p* = 0.01) and have right heart failure and while not statistically significant, it had a higher estimated mean PAP on echocardiography (*p* = 0.06). The group that received the surgical procedure tended to have a higher mean Cr level. This clearly differentiates therapies as to the pathophysiologic mechanism behind the development of the clinically symptomatic pericardial effusion in SSc. The surgical group which tended to be younger had a mean Cr level greater than the medically treated group which suggests a uremic type of pericardial effusion, less associated with PAH and more likely to proceed with surgical management when this was warranted. In contrast to the medical management group, while having similar size pericardial effusions happened to be associated with higher PAP and right heart failure and the clinicians were less likely to use a surgical approach. This may relate to the fact that clinicians are aware that, in PAH patients who have a pericardial effusion a pericardiocentesis can lead to acute right heart decompensation and thus choose medical management instead. Of note, Maione et al. reported 47% of patients with scleroderma showed PAP > 30 mmHg. SSc patients with PAH and large pericardial effusions should have a right heart catheterization to evaluate in detail the hemodynamics of the right heart before even a pericardial surgical procedure is contemplated. In contrast patients who develop pericardial disease from other causes other than right heart failure, such as renal disease, may be more amenable to a surgical procedure and thus the clinical teams are more likely to recommend.

Limitations of this study include the small number of patients included due to the rarity of the disease, the retrospective nature of the study, and the incomplete clinical data. Also the size description of the pericardial effusion is clinician-dependent. This is the largest cohort of SSc patients with clinically symptomatic pericardial effusions who were studied in the inpatient setting and our data suggest that the pathophysiologic mechanism of the pericardial effusion is taken into consideration when clinical decisions are to be made.

## Figures and Tables

**Table 1:** Characteristics of all SSc patients admitted for pericardial disease.

	All patients *n* = 23
Sex	19 F, 4 M
Age	60 (range: 44–79)
Race	14 C, 5 AA, and 2 unknown
SSc type	LcSSc 6, dcSSc 6, and unknown 11
Right heart failure	11 (48%)
Tamponade	4 (17%)
PAH	17 (74%)
Medical/surgical therapy	18/5
Readmission	3
Death during admission	2
Death up to 2/2017	8

F = female; M = male; C = Caucasian; AA = African American; LcSSc = limited systemic sclerosis; dcSSc = diffuse systemic sclerosis; PAH = pulmonary arterial hypertension.

**Table 2:** Individual clinical characteristics of SSc patients with pericardial disease.

Patient	Age	Type of SSc	Diagnosis	Overlap syndrome	Size of pericardial effusion	Presence of tamponade	Right heart failure	Procedure	Fluid study	Meds prior to admission
1	60	Unknown			M-L	-	Severe			
2	71	Limited		+ (PM, MCTD)	M	-	Mild	Thoracentesis	Pleural fluid: transudative (LDH 88, Pr 1.3, NUC 63, seg 52)	
3	60	Unknown			L	-	Severe			Iloprost, sildenafil, bosentan, and plaquenil
4	68	Unknown			M-L	-				
5	62	Limited	New		M-L	+	Severe			
6	61	Diffuse			M-L	-				Methylprednisolone
7	55	Limited			M	-	Mild			Treprostinil, macitentan, sildenafil
8	65	Unknown			S	-				Mycophenolate, tacrolimus, and prednisone
9	79	Unknown			L	-	Severe			
10	66	Limited	28 yrs		M	-				Ambrisentan, nifedipine
11	57	Unknown			M	-				Nifedipine
12	49	Unknown			M	-				
13	72	Unknown	New		M	-	Moderate			Nifedipine, ibuprofen
14	59	Unknown	New	+ (IBM)	M-L	-				
15	63	Limited	2 yrs	+ (Sicca)	M	-	Severe			Cevimeline, candesartan
16	58	Unknown	13 yrs		L	-	Moderate			Ambrisentan, sildenafil, iloprost, and mycophenolate
17	57	Unknown	14 yrs		M-L	+	Severe	Thoracentesis		Macitentan, mycophenolate, and sildenafil
18	56	Limited			L	+	Mod-severe			Sildenafil
19	56	Diffuse	2 mo		L	-		Pericardiocentesis	WBC 178, RBC 9, GS neg, Cx neg, cytology neg	Methotrexate, prednisone
20	48	Diffuse	8 yrs	+ (MCTD)	L	-		Pericardiocentesis → pericardial window	WBC 240, RBC 3550, pleural fluid: no malignancy, reactive mesothelial cells, and chronic inflammation	Lisinopril
21	44	Diffuse	New		L	-		Pericardiocentesis → pericardial window	Cytology neg, AFB neg	
22	66	Diffuse	New		L	+		Pericardiocentesis → pericardial window	WBC 568, RBC 815, GS neg, and cytology neg	Prednisone
23	45	Diffuse	7 mo	+ (RA, Sjogren, DM)	M	-		Pericardial window		Methotrexate, plaquenil

S = small, M = moderate, L = large, PM = polymyositis, IBM = inclusion body myositis, MCTD = mixed connective tissue disease, RA = rheumatoid arthritis, and DM = dermatomyositis.

**Table 3:** Admission demographics, presenting symptoms, workups, and outcomes by subgroup.

	Medical therapy (*n* = 18, 22 admissions)	Procedure (*n* = 5, 10 admissions)	
*Demographics*			
Sex	15 F, 3 M	4 F, 1 M	*p* = 1.00
Age	62.1 (7.1)	51.8 (9.2)	**p** = 0.01
Race	12 C, 3 AA, 2 other, and 1 unknown	2 C, 2 AA, and 1 unknown	
Limited versus diffuse	6 limited, 1 diffuse, and 11 unknown	5 diffuse	

*Scleroderma disease characteristics*			
Raynaud phenomenon	6	2	
Digital ulcers	1	0	
Interstitial lung disease	5	2	
Esophageal involvement	2	0	

*Symptoms*			
Shortness of breath	12	3	
Chest pain	2	0	
Fever	2	0	

*Workup*			
Reported hypoxia	7	0	
Pericardial effusion size	1 small, 12 mod-large, 5 large	1 mod, 4 large	
Right heart failure	11	0	
Tamponade physiology	2	2	
Pulmonary artery hypertension	14 (2 unknown)	3	*p* = 0.22
PASP by echo (mmHg)	79.8 ± 45	39.7 ± 11	*p* = 0.06
WBC (10^9^/L)	10 ± 5.2	8.7 ± 4.8	*p* = 0.56
Hgb (g/dL)	11.6 ± 2.1	10.3 ± 2.8	*p* = 0.26
Plt (10^9^/L)	292 ± 126	275 ± 150	*p* = 0.79
Cr (mg/dL)	1.2 ± 0.51	2.6 ± 2.5	**p** = 0.03
BUN (mg/dL)	22.6 ± 7.7	27.5 ± 31	*p* = 0.53
AST (U/L)	17.4 ± 6.5	15.5 ± 2 (2 patients)	
ALT (U/L)	25.2 ± 9.1	37.5 ± 12 (2 patients)	
proBNP (pg/ml)	4671.6 ± 4011	338.5 ± 269	*p* = 0.15
ANA	10 pos, 2 neg	4 pos, 1 neg	
ANA pattern	2 nucleolar, 4 centromeres, 2 speckled, 1 diffuse, and 1 unknown	2 nucleolar, 1 speckled, and 1 diffuse	
Scl-70 Ab	12 neg	4 neg	
ESR (mm/hr)	51.6 ± 31	None reported	
CRP (mg/dL)	47 ± 46	1.9 (1 patient)	

*Outcomes*			
Readmission in 90 days	3 readmissions in 2 patients	1	
Death (as of 2/2017)	7 (2 during the admission)	1	
